# Misalignment Assembly Effect on the Impact Mechanical Response of Tandem Nomex Honeycomb-Core Sandwich Structures

**DOI:** 10.3390/ma17164024

**Published:** 2024-08-13

**Authors:** Yufan Yin, Xiaojing Zhang

**Affiliations:** School of Aeronautics and Astronautics, Shanghai Jiao Tong University, Shanghai 200240, China; yyfyyfyyf@sjtu.edu.cn

**Keywords:** tandem honeycomb, misalignment assembly, mechanical response, finite element model, failure behavior

## Abstract

To optimize the assembly methods of honeycomb structures and enhance their design flexibility, this study investigated the impact mechanical responses of tandem honeycomb-core sandwich structures with varying misalignment assembly lengths. Impact tests were conducted across different energy levels on single-layer and tandem honeycomb-core sandwiches to observe their impact processes and failure behaviors. Our findings indicate that tandem honeycomb cores significantly enhance the impact resistance compared with single-layer configurations, even though a misaligned assembly can deteriorate this property. A finite element model was developed and validated experimentally; the model showed good agreement with the experiments, thereby allowing the simulation and evaluation of the impact responses. Herein, we reveal that specific misalignment lengths can either increase or decrease the impact resistance, providing insights into improving the resilience of tandem honeycomb-core structures. Our results not only contribute to enhancing the impact resistance of honeycomb-core sandwich structures but also offer a valuable basis for their practical applications in engineering.

## 1. Introduction

Composite materials are highly implemented in industry and research because of their excellent specific strength, stiffness, and design flexibility. This is particularly true in the aerospace industry, where lightweight structures are crucial [1,2,3,4,5,6]. Composite sandwich structures are a unique type of composite that are lightweight and offer excellent specific flexural stiffness [7], thermal insulation [8], and acoustic damping [9]. These properties provide significant advantages in engineering applications [10,11,12,13,14,15], which has led to their widespread use in marine, aerospace, and automotive manufacturing as well as mechanical engineering [16,17,18,19,20]. However, in practical applications, composite sandwich structures are susceptible to impacts from runway debris, hail, and dropped tools, among other objects, which can result in the delamination and local fractures of face sheets [21,22,23,24,25]. Such damage can significantly reduce the load-bearing capacity and compromise structural integrity [26]. Thus, increasing attention is being drawn to the impact performance of these structures.

Honeycomb cores are preferred in fuselage structures owing to their superior stability [27]. The failure mode of honeycomb sandwich structures depends on the face sheet thickness, cell size, and wall thickness [28]. Potential damage includes fiber fracture, delamination, debonding, core crushing, shearing, and matrix cracking [29]. At low-impact energies, a honeycomb core primarily undergoes buckling and wall folding [30]. As the impact energy increases, core crushing becomes predominant [31]. In particular, Xie [32] found that a lower core density in Nomex honeycombs increases the absorbed energy, whereas higher-density cores exhibit greater peak loads [33]. The results provided by Li [34] and Xiang [35] suggest that increasing the number of honeycomb layers, cell wall thickness, and cell count can improve the energy-absorption efficiency, with out-of-plane gradient designs further enhancing this efficiency.

The impact performance of single-layer honeycomb sandwich structures is well-understood. Tandem honeycombs can provide greater compression strokes and enhance the performance of functional materials between the layers. They offer increased strength, energy absorption, multifunctionality, and lightweight designs, making them popular in engineering applications [36]. Increasing the number of core layers improves the impact resistance [37,38,39,40] and energy-absorption rates [41] of sandwich structures. Sun et al. [42] studied multilayer circular honeycombs under in-plane impacts and found that the optimal energy absorption was linked to the dynamic plateau stress and densification strain, with the optimal energy-absorption efficiency being the inverse of the dynamic densification strain. Giulia et al. [43] analyzed the energy-absorption capacity of single-layer and double-layer aluminum honeycomb structures and showed that double-layer panels exhibited a progressive collapse sequence influenced by the core arrangement and cell size. Honeycombs with larger cell sizes demonstrated a more even distribution of impact loading, resulting in the almost uniform compression of the core. Zhang [44] investigated multilayer curved aluminum honeycomb beams, highlighting that the core thickness and the upper and lower core thicknesses—notably affect deformation, failure modes, and energy absorption. Yan [45] observed that double-layer configurations absorbed considerable energy in the upper layer, reducing back-panel deflection. Fan et al. [46] concluded that gradient-tandem honeycomb panels outperformed uniform-tandem panels in terms of impact displacement. During honeycomb structure assembly, errors frequently occur because of manual operations, manufacturing equipment limitations, or material variations. These unavoidable factors produce minor deviations in alignment and cause the imperfect assembly of structures. These deviations include cell misalignments, size discrepancies, and positional shifts. Although each deviation is seemingly minor, these discrepancies accumulate with increased assemblies and can significantly affect the overall performance and characteristics of a structure. Therefore, studying the mechanical properties of misaligned honeycomb assemblies is crucial for optimizing material performance, refining assembly processes, and providing reliable, practical guidance. Table 1 summarizes the structural similarities, differences, and advances of this study and those described above regarding the impact on honeycomb structures, particularly with respect to the tandem honeycomb structure.

## 2. Experiment

### 2.1. Specimen Preparation

**Figure 1:**
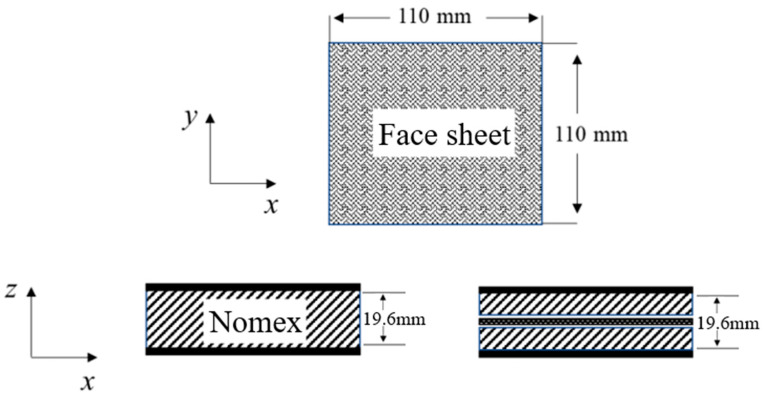
Nomex honeycomb sandwich-structure impact specimen.

**Figure 2:**
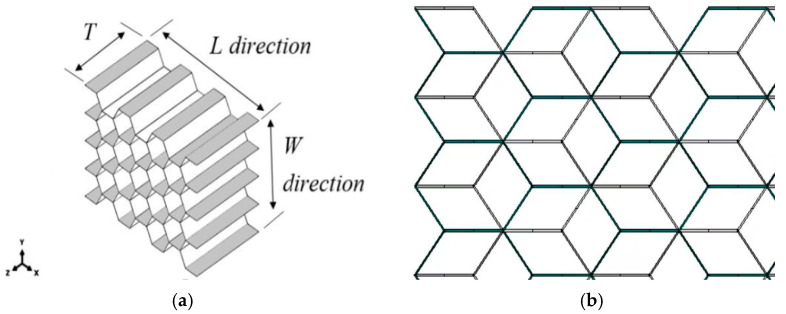
Nomex honeycomb structure: (**a**) Dimension and direction of the honeycomb core; (**b**) Top view of a misaligned tandem honeycomb-core structure.

The specimens used in the experiments are illustrated in Figure 1. The specimens’ dimensions are 110 mm × 110 mm; the epoxy resin glass fiber prepreg EV101-6/7581 was selected to lay-up the upper and lower panels. This prepreg consists of high-strength glass fibers pre-impregnated with epoxy resin, ensuring uniform resin distribution and optimal fiber–matrix adhesion. The lay-up order was [45/−45]. The prepreg was cut into the required size and shape for the specimens. The core was made of regular hexagonal Nomex honeycombs with a cell size of 3.2 mm, a lattice edge length of 1.85 mm, and a core density of 48 kg/m^3^. These honeycombs were fabricated from Nomex aramid paper with a nominal thickness of 0.05 mm. Due to the manufacturing process, the cell walls in the L direction (shown as white in Figure 2b) were double compared to the other cell walls. Table 2 shows a comparison of the specimen groups. The specimens included a single honeycomb sample (C), two identical honeycomb layers separated by microstructure interface layers in an aligned assembly (CW), and two identical honeycomb layers separated by microstructure interface layers in a misaligned assembly (CWC). A wave-absorbing film material was used as the interface of the honeycomb layer spacer, and the film substrate was a polyimide insulating material. “Aligned assembly” indicates that the upper and lower honeycomb layers were assembled without overlapping cells, whereas “misaligned assembly” implies that one honeycomb core was shifted by one cell length along the L direction, resulting in hexagonal corner point support between layers. Figure 2a,b show the honeycomb-core direction and top view of the misaligned tandem honeycomb-core structure, respectively.

### 2.2. Impact Experiment

**Figure 3:**
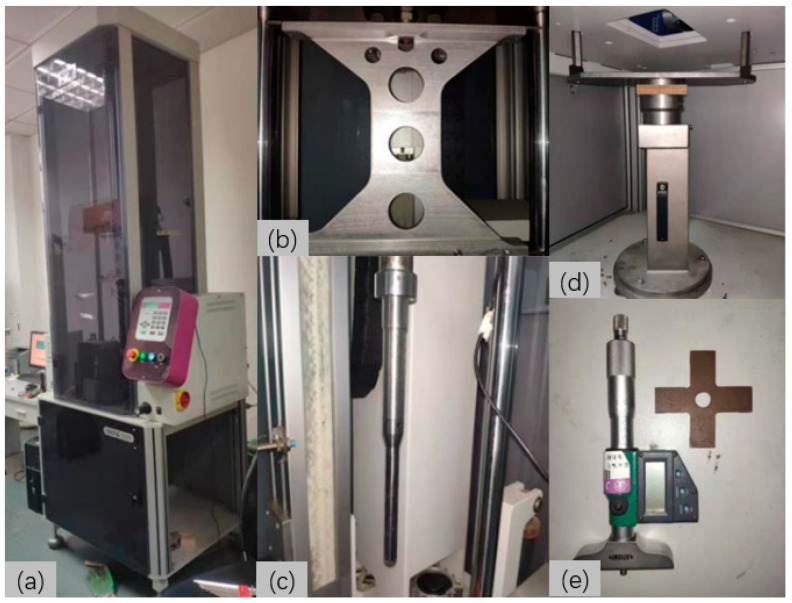
Test instrumentation and setup: (**a**) Drop-weight impact tester; (**b**) Drop-weight frame; (**c**) Impactor; (**d**) Fixture; (**e**) Indentation depth gauge.

The experiment was conducted according to the ASTM-D3763 standard [47]. An INSTRON CEAST 9350 drop-weight impact tester was used (Figure 3a). The upper part of the impact tester included the control system, a drop-weight frame (Figure 3b), an impactor (Figure 3c) with a diameter of 12.7 mm and mass of 2.131 kg, and a photoelectric speed gate. The lower part contained the experimental fixture and specimen. A standard ring-shaped fixture with an inner diameter of 76 mm and an outer diameter of 100 mm was used (Figure 3d). The specimens were divided into three groups: control (C), CW, and CWC. For the nonpenetrated specimens, the indentation depth was measured using a 3540-B25S indentation depth gauge (Figure 3e), following the ASTM-D7136 standard [48]. Data on the impact time, speed, deformation, and energy were collected at 100 kHz.

## 3. Experimental Results

### 3.1. Force–Time Curves and Force–Deformation Curves after Impact

#### 3.1.1. Single-Layer Honeycomb

**Figure 4:**
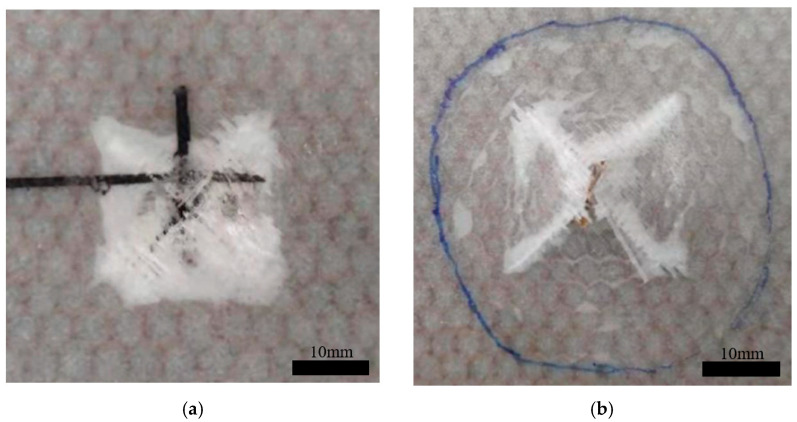
Typical damage patterns of the upper (**a**) and lower (**b**) face sheets in the C group after perforation.

In the single-layer honeycomb specimens, impact tests were conducted at different energy levels. When the energy reached 16 J, the structure was completely penetrated, and no further increase in energy was attempted. As the impact energy increased, the damage to the sandwich structure ranged from the simple delamination of the face sheet to complete penetration, as shown in Table 3. At impact energies of 2 J and 4 J, only the upper face sheet was damaged. When the impact energy reached 8 J, the upper face sheet exhibited ±45° cross cracks and was completely penetrated. At 12 J, both the upper and lower face sheets were affected. At 16 J, the entire structure was completely penetrated. The typical damage modes of the upper and lower face sheets in the C group after penetration are shown in Figure 4, where ±45° fiber fracture traces corresponding to the material lay-up direction can be observed.

**Figure 5:**
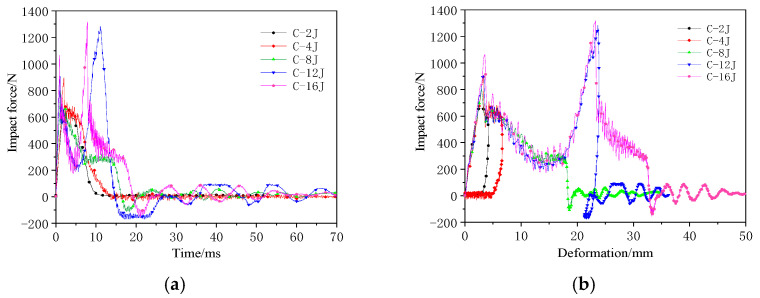
Impact mechanical response curves of the single-layer honeycomb-core structure: (**a**) Impact force–time curve; (**b**) Impact force–deformation curve.

The impact force–time and impact force–deformation curves for the single-layer honeycomb-core structure during the impact test are shown in Figure 5.

At low-impact energy levels, the impact force response curve exhibited only a single peak. However, when the impact energy was sufficient to affect the lower panel, the curve exhibited two distinct peaks. In the impact force–deformation curve, the deformation represents the displacement of the impactor. At low-impact energies, the panels are not penetrated, and the impactor rebounds, resulting in hysteresis in the low-energy response curves. When the energy was sufficiently high to penetrate the panels, the impactor moved deeper into the core, facing resistance from the core, which produced a plateau in the response curve (C-8 J response), with a plateau stress of approximately 300 N. With a further increase in energy, the impactor reached the lower panel, and the impact force–deformation curve showed a second peak. The measured data indicated that the second peak force was significantly higher than the first peak. The positions of the two peak forces were approximately 20 mm apart, corresponding to the thickness of the specimen. The curves indicate that the stiffness of the impact response remained similar across all energy levels.

#### 3.1.2. Double-Layer Aligned Honeycomb

In the double-layer aligned assembly CW group specimens, the presence of a thin-film interlayer increased the resistance. The impact tests considered the same energy levels as those for the C group. At 16 J, the lower panel of the CW group presented a slight delamination. Therefore, the energy was increased to 20 J to achieve complete penetration.

**Figure 6:**
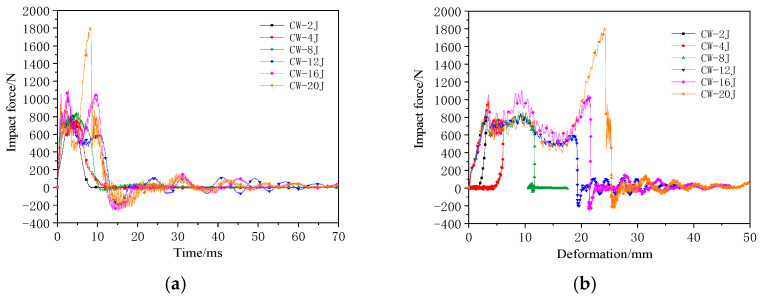
Impact mechanical response curves of the double-layer aligned honeycomb-core structure: (**a**) Impact force–time curve and (**b**) impact force–deformation curve.

The impact force–time and impact force–deformation curves of the double-layer aligned honeycomb structure during the impact tests are shown in Figure 6. Before the panels were penetrated, the impact force exhibited a linear relationship. At 8 J, the upper panel of the CW group was not fully penetrated, as was the case with the C group. However, the CW group exhibited significant fiber breakage damage, rapidly reducing the impact force without a plateau phase. Table 4 lists the impact parameters and results.

**Figure 7:**
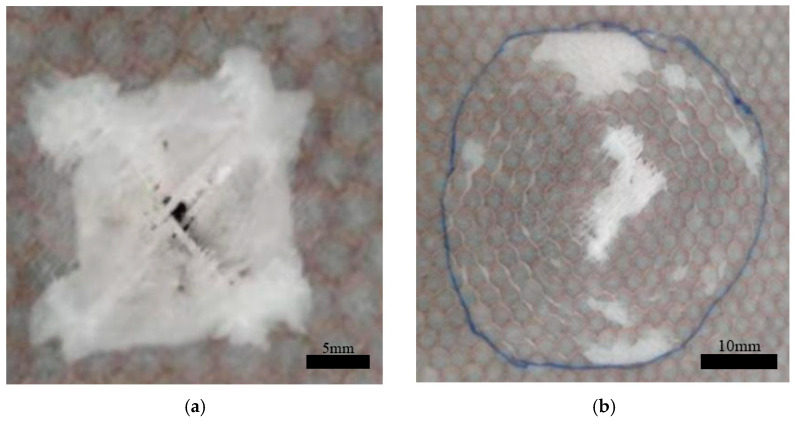
Typical damage patterns of the upper (**a**) and lower (**b**) face sheets in the CW group after perforation.

At 12 J, the impactor breaks through the upper panel, enters the core, and penetrates the film, resulting in a stable phase. The stable contact force for the CW group was approximately 500 N, exhibiting an increase of over 66% compared with that of the C group. At 16 J, the impactor damaged the lower panel, which resulted in slight delamination. The second peak force was slightly higher than the first peak force. At 20 J, the lower panel exhibited large-area delamination with significant fiber breakage and noticeable cracks at the impact center. The impactor did not fully penetrate the lower panel, and the second peak force was almost twice the first peak force. The distance between the two peak forces was approximately 20 mm, which is consistent with the specimen thickness. Figure 7 shows the typical upper and lower panel damage modes for the CW group at 20 J.

#### 3.1.3. Double-Layer Misaligned Honeycomb

For the misaligned assembly of the CWC group specimens, impact tests were conducted using the same energy levels as those of the CW group. The test results are listed in Table 5.

**Figure 8:**
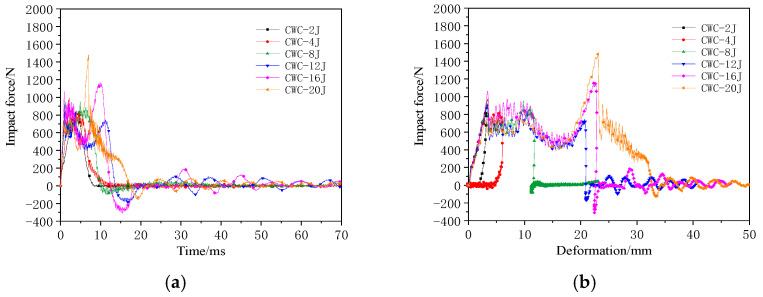
Impact mechanical response curves of the double-layer misaligned honeycomb-core structure: (**a**) Impact force–time curve; (**b**) Impact force–deformation curve.

The impact force–time and impact force–deformation curves of the misaligned tandem honeycomb sandwich structure are shown in Figure 8.

**Figure 9:**
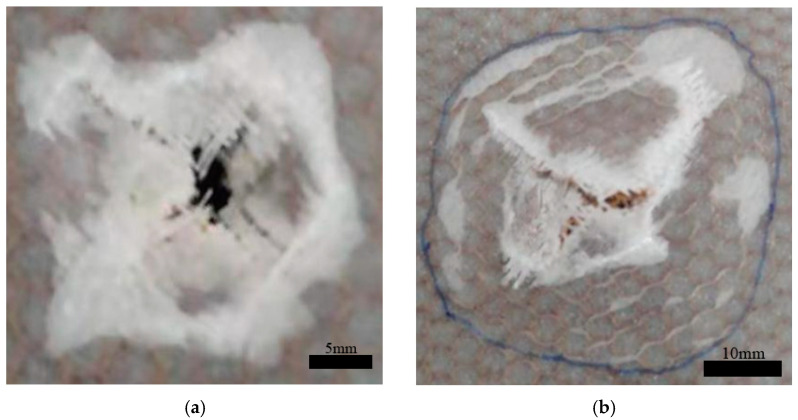
Typical damage patterns of the upper (**a**) and lower (**b**) face sheets in the CWC group after perforation.

The impact force response of the CWC group was similar to that of the CW group. At 20 J, the lower panel was completely penetrated, with ±45° cracks appearing at the impact center with a large delamination area around it. The second peak force was 1.5 times the first peak force. The response distance between the two peak forces was approximately 20 mm, which matched the thicknesses of the specimens. Figure 9 shows the typical damage modes in the upper and lower panels of the CWC group at 20 J.

### 3.2. Energy-Absorption History after Impact

**Figure 10:**
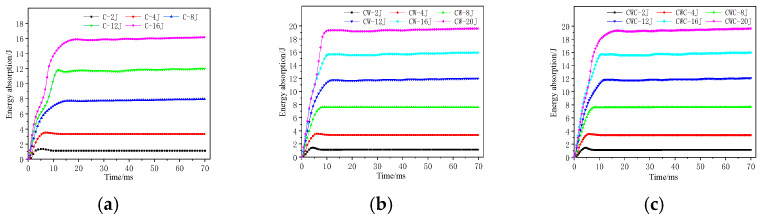
Energy-absorption–time history curves of different honeycomb structures: (**a**) C group; (**b**) CW group; (**c**) CWC group.

The energy absorption of the honeycomb sandwich structure can be obtained by integrating the force–deformation curve, as shown in Figure 10. The time required for the energy-absorption curve to increase to its maximum value indicates that the greater the impact energy, the longer the contact time between the impactor and structure, the deeper the penetration into the structure, and the longer the energy-absorption time. During low-energy impacts, the curve exhibits a slight downward trend after reaching its maximum value due to the rebound of the impactor, which did not penetrate the structure completely. As the impact energy increases, the impactor penetrates the structure without rebounding, reducing or even eliminating the downward trend.

## 4. Comparisons and Discussion

### 4.1. Effect of Dislocation on the Impact Mechanical Response of a Tandem Honeycomb

The impact response of the honeycomb sandwich structure was evaluated in terms of the contact force, impact displacement, and energy absorption. The first peak of the contact force reflected the resistance of the front panel of the sandwich structure. Therefore, the first peak force and impact displacement were used as the evaluation indicators. The changes in impact resistance for each group are listed in Table 6 and Table 7, where the amount of change refers to the change compared with group C.

**Figure 11:**
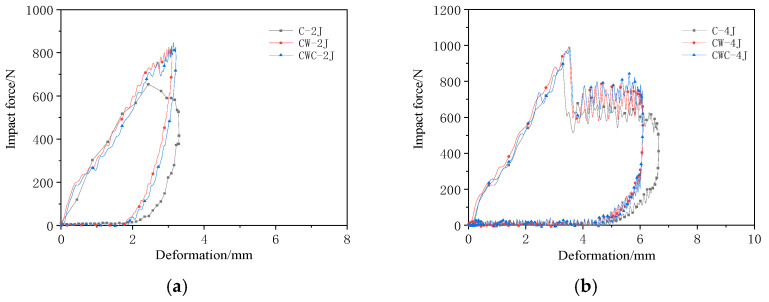
Comparison of the impact mechanical responses of the different configurations at low impact energies of (**a**) 2 J and (**b**) 4 J.

Figure 11 shows a comparison of the impact mechanical responses of the three structural groups under low-impact energy. Based on the results shown in Table 6 and Table 7, under 2 and 4 J, the front panels of all three configurations did not penetrate, and the response forms of the CW and CWC groups were consistent. At an impact energy of 2 J, the first peak forces of the CW and CWC groups were 26.34% and 29.41% higher than those of the C group, respectively. At an impact energy of 4 J, the first peak forces for the CW and CWC groups were 993.5 and 990.7 N, respectively, and both were approximately 11% higher than the 895.4 N of the C group. At an impact energy of 2 J, the impact displacement of the CW group was 3.10 mm, which was 6.01% less than the 3.30 mm of the C group, whereas the CWC group was 3.21 mm, which was 2.60% less than that of the C group. At an impact energy of 4 J, the impact displacements for the CW and CWC groups were 6.10 mm and 6.09 mm, respectively, which were approximately 8% less than the 6.64 mm of the C group.

The impact response results for the C group indicated that the single-layer honeycomb structure had a poorer impact resistance. The impact peak force was approximately 30% lower than that of the double-layer honeycomb structure. Under a low-impact energy, the impactor required a greater deformation to stop, indicating that the structure deformed more easily under the impact, severely damaging the panel. Thus, the double-layer series structure design demonstrated improved impact resistance under a low-impact energy.

In summary, the double-layer honeycomb sandwich structures exhibited greater resistance and less deformation under low-impact energy conditions, effectively protecting the panels and performing significantly better than the single-layer honeycomb structure.

**Figure 12:**
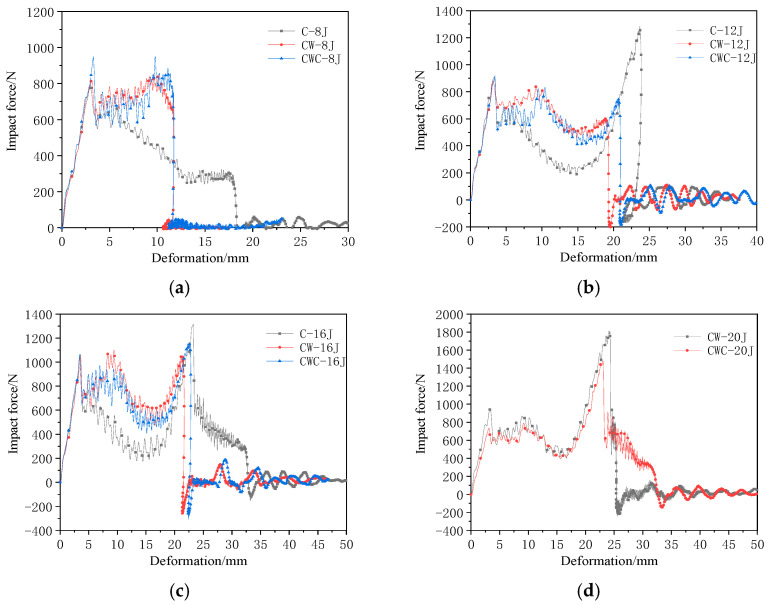
Comparison of the impact mechanical responses of the different configurations at high energies: (**a**) 8 J; (**b**) 12 J (**c**) 16 J; (**d**) 20 J.

Because the upper panel of the single-layer honeycomb of the C group penetrated at 8 J, we defined impact energies above 8 J as high-impact energies. The comparison results of the mechanical responses are shown in Figure 12. At impact energy of 8 J, the first peak forces for the CW and CWC groups were 853.9 and 950.4 N, respectively, representing increases of 6.74% and 18.80%, respectively, compared to that of Group C (800 N). Both the CW and CWC groups exhibited cracks on the upper panel without complete penetration, resulting in extended contact time and a plateau in the contact force curve. In contrast, Group C showed a rapid decrease in the contact force after reaching its peak because of the upper panel penetration. The impact displacement for the C group was 18.31 mm, whereas the CW and CWC groups exhibited displacements of 11.71 and 11.74 mm, respectively, indicating reductions of 36.05% and 35.88% compared with that of the C group. At an impact energy of 12 J, the impactor displacement for the CWC group was 20.93 mm, which was 6% greater than the 19.31 mm of the CW group, indicating the improved impact resistance of the honeycomb core of the CW group. At an impact energy of 16 J, the C group was completely penetrated, with the frictional resistance from the panels causing fluctuations in the response curve. This was followed by a rapid decline. The lower panels of the CW and CWC groups also affected the energy. The CWC group showed a 4% higher stopping displacement than that of the CW group, indicating more significant irreversible damage to the lower panel. The contact force of the CW group was higher during the plateau phase, indicating a better core resistance. At 20 J, both panels of the CWC group were completely penetrated, and the response curve showed typical penetration fluctuations. In contrast, the CW group exhibited significantly less panel cracking without complete penetration. The first peak force for the CW group was 945.4 N, which, compared to the 789.5 N of the CWC group, indicates the higher impact resistance of the CW group. Moreover, the second peak force was also higher in the CW group, indicating better resistance at high-impact energies.

**Figure 13:**
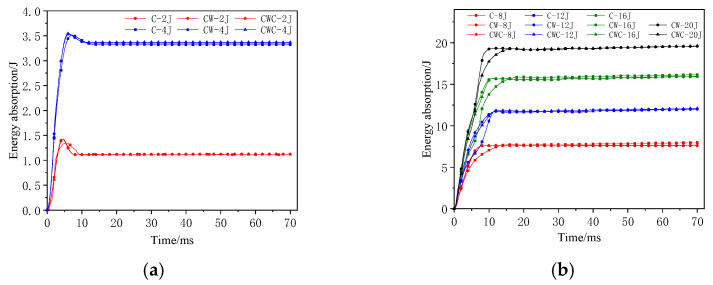
Energy-absorption–time history curves of honeycomb structures at (**a**) low and (**b**) high energies.

Figure 13 shows the energy-absorption curves over time for the three structures at different impact energies. The double-layer honeycomb structures significantly reduced the energy-absorption time under high-energy impacts, whereas the misalignment minimally affects the absorption time.

In conclusion, the double-layer honeycomb structures exhibited superior energy absorption compared with the single-layer honeycomb structures at the same impact energy, displaying shorter impactor displacement and less irreversible damage to the panels. The penetration energy of the upper panel for the single-layer honeycomb structures was lower than that for the double-layer series honeycomb structures. Among the double-layer structures, the aligned honeycomb cores have improved impact resistance than the misaligned cores. Misaligned structures require a greater impactor displacement to entirely absorb the impact energy, which reduces their impact resistance. Overall, misalignment negatively affected the impact resistance of the series of honeycomb structures.

### 4.2. Finite Element Simulations and Validation

**Figure 14:**
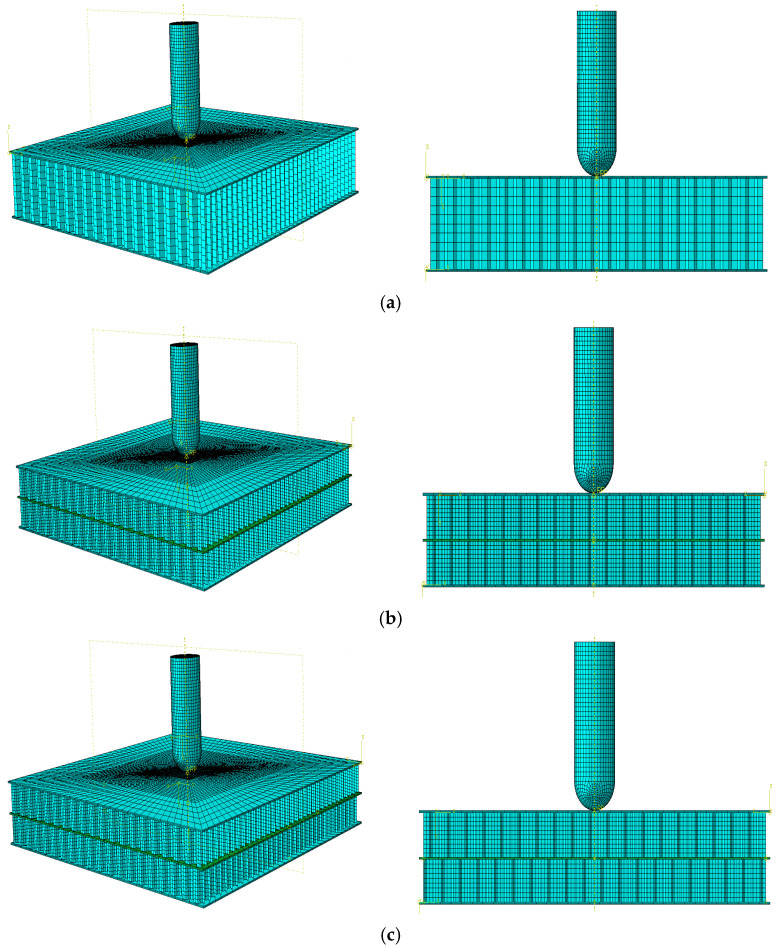
Finite element models of honeycomb structures: (**a**) Single-layer honeycomb; (**b**) double-layer aligned honeycomb; (**c**) double-layer misaligned honeycomb.

A finite element model was developed based on the geometric dimensions of the composite sandwich structure during the actual tests, as shown in Figure 14.

A finite element simulation was conducted using ABAQUS. The panels were divided into two grids according to the thickness direction, with each grid representing a layer. Grid refinement was conducted in the area subjected to impulse, with the size of the refined element being 0.5 mm × 0.5 mm. The panels were modeled using element type C3D8R, considering the material parameters listed in Table 8. S4R was chosen as the element type for the honeycomb core. A total of 220,131 elements were included, of which 149,322 were core elements and 70,809 were panels. The impactor was defined as a rigid body with a mass of 2.131 kg, corresponding to the experimental value. The thin film layer was treated as a bilinear material using a bilinear ontological model. A cohesive element was created to characterize the thin film layer by offsetting mesh; the material properties of the cohesive were obtained from experimental data, including Quads Damage and Damage Evolution. The specific parameters are presented in [27]. The actual thickness of the cohesive layer was set in the cross-section part, and its contact with the honeycomb-core layer was set by specifying the upper and lower surfaces of the cell. The element type was COH3D8. The central region mesh of the panel was refined, and the hourglass control was enhanced for stability. A “Tie” constraint was used between the panels and the core to simplify the model by reducing computation time.

Two contact types were established. Surface-to-surface contact was used for the impactor and panel, within the panel and between the honeycomb layers, and general contact properties were used for all the other contacts. The normal behavior was set to “hard” contact, and the tangential behavior was set to “penalty” friction with a coefficient of 0.1. Fixed boundary conditions were applied to the edges of the upper and lower panels, constraining all the degrees of freedom (U1 = U2 = U3 = R1 = R2 = R3 = 0). The reference point was tied to the impactor with only three directions free, whereas the other degrees of freedom were constrained (U1 = U2 = R1 = R2 = R3 = 0). The initial impact velocities in the three directions were set using a predefined field function to define the different energy impact loads.

To characterize the damage modes of the panels, the vumat subroutine was manually written. The Hashin failure criterion for three-dimensional solid elements was employed, considering the failure modes of composite materials to include the fiber tensile failure, fiber compressive buckling fracture, matrix tensile failure, and matrix compressive cracking failure as follows:

Fiber tensile failure ($\sigma_{11}\geq 0$):

$${\left(\frac{\sigma_{11}}{S_{11}^{T}}\right)}^{2}+{\left(\frac{\tau_{12}}{S_{12}}\right)}^{2}+{\left(\frac{\tau_{13}}{S_{13}}\right)}^{2}\geq 1$$

Fiber compressive failure ($\sigma_{11}<0$):

$${\left(\frac{\sigma_{11}}{S_{11}^{C}}\right)}^{2}\geq 1$$

Matrix tensile failure ($\sigma_{22}+\sigma_{33}\geq 0$):

$${\left(\frac{\sigma_{22}+\sigma_{33}}{S_{22}^{T}}\right)}^{2}+\frac{1}{S_{23}^{2}}\left(\tau_{23}^{2}-\sigma_{22}\sigma_{33}\right)+{\left(\frac{\tau_{12}}{S_{12}}\right)}^{2}+{\left(\frac{\tau_{13}}{S_{13}}\right)}^{2}\geq 1$$

Matrix compressive failure ($\sigma_{22}+\sigma_{33}<0$):

$$\frac{\sigma_{22}+\sigma_{33}}{S_{22}^{C}}\left[{\left(\frac{S_{22}^{C}}{{2S}_{23}}\right)}^{2}-1\right]+\frac{{\left(\sigma_{22}+\sigma_{33}\right)}^{2}}{{4S}_{23}^{2}}+\frac{\tau_{23}-\sigma_{22}\sigma_{33}}{S_{23}^{2}}+{\left(\frac{\tau_{12}}{S_{12}}\right)}^{2}+{\left(\frac{\tau_{13}}{S_{13}}\right)}^{2}\geq 1$$

**Figure 15:**
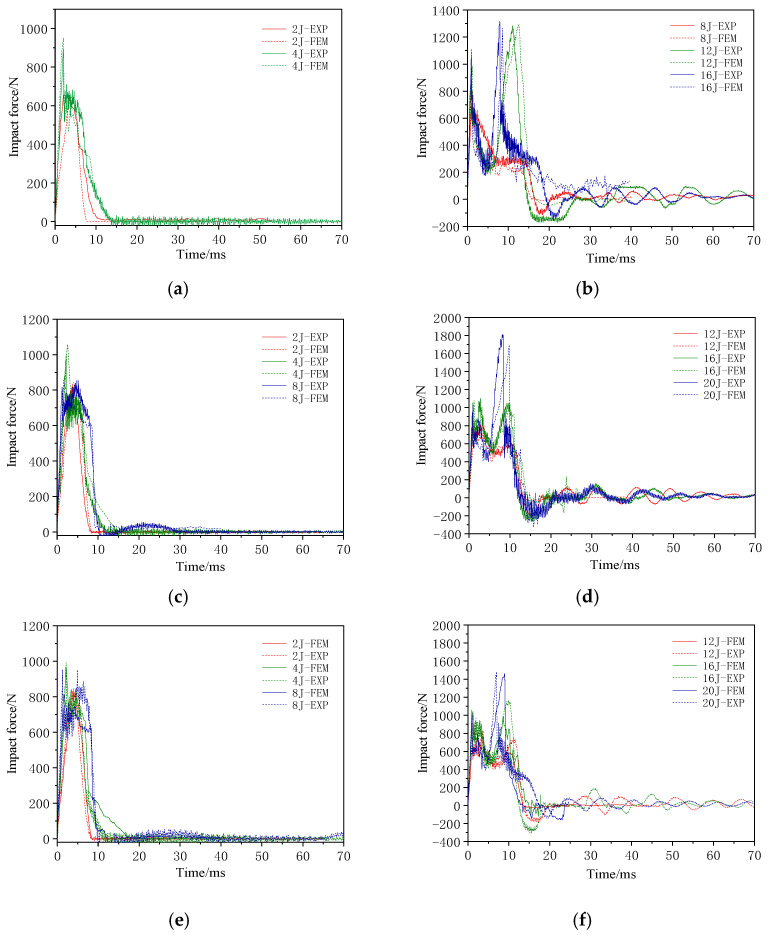
Impact force–time curves from experiments and finite element results for the different honeycomb structures: (**a**) C group at low energy; (**b**) C group at high energy; (**c**) CW group at low energy; (**d**) CW group at high energy; (**e**) CWC group at low energy; (**f**) CWC group at high energy.

**Figure 16:**
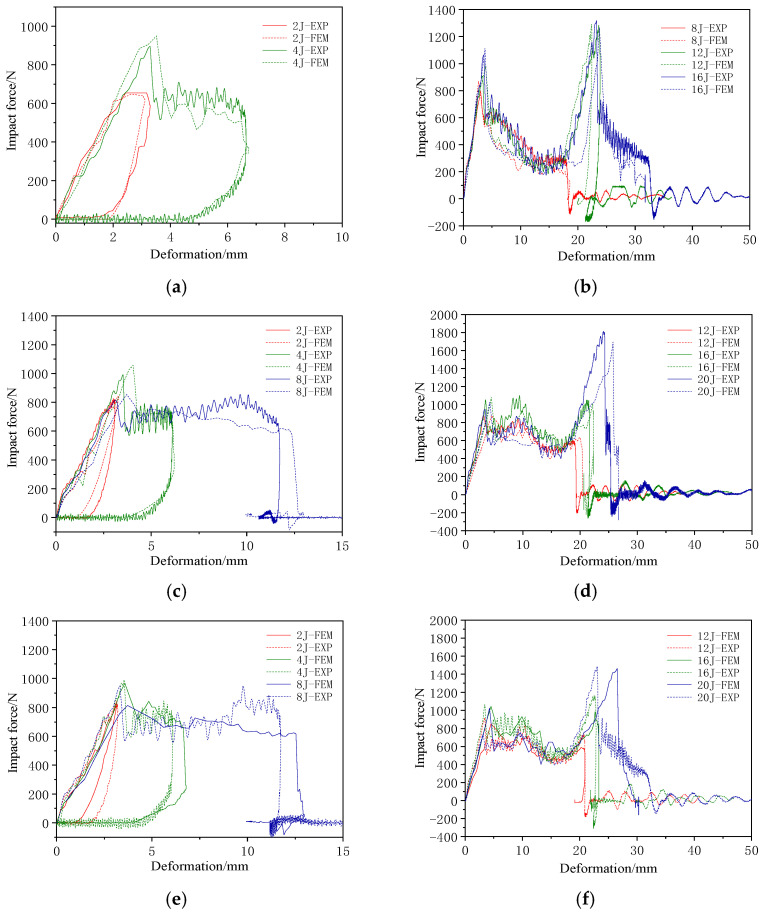
Impact force–deformation curves from experiments and finite element results for the different honeycomb structures: (**a**) C group at low energy; (**b**) C group at high energy; (**c**) CW group at low energy; (**d**) CW group at high energy; (**e**) CWC group at low energy; (**f**) CWC group at high energy.

Figure 15 and Figure 16 present a comparison of the force–time and force–deformation curves from the finite element simulations and experimental results for the three structures under different impact loads. Table 9 and Table 10 show a comparison of the experimental and finite element results of first peak forces and displacement magnitudes. The curve trends of the finite element results are consistent with the experimental results, and the errors in the peak force and displacement data are controlled within approximately 10%. Although the error in the first peak force for the CWC group under an impact energy of 20 J was relatively large, it remained low in most cases, demonstrating the reliability of the finite element simulation in predicting the peak forces. Therefore, the finite element results were in good agreement with the experimental results.

In the material parameter subroutine for the panels, the state variables were defined as SDV1, SDV2, SDV3, and SDV4, representing fiber tension, fiber compression, matrix tension, and matrix compression, respectively. The damage types and areas of the upper and lower panels after impact were extracted and are listed in Table 11, Table 12 and Table 13. Cross-sectional images of the specimens after impact are shown in Table 14. The accuracy of the finite element model was verified by comparing the results with the experimental data.

### 4.3. Effects of Different Dislocation Distances on the Mechanical Responses of Tandem Honeycombs

Misalignment is a significant influencing factor introduced in this study. This misalignment is challenging to avoid when manufacturing serially connected honeycombs. Previous studies have only focused on the CWC group, which is a specific misalignment mode. This misalignment mode was selected because it uses honeycomb cell edges and vertices as positional reference points during assembly, which helps reduce assembly errors and maintains the double-layer honeycomb walls in the same plane. Compared to a single-layer wall region, this setup provides improved support and avoids any significant performance degradation of the structure.

**Figure 17:**
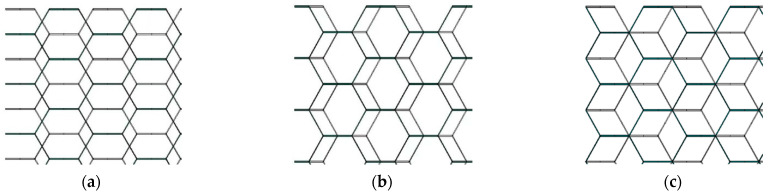
Top views of honeycomb structures with different misalignment distances: (**a**) CWC-3/2; (**b**) CWC-1/2; (**c**) CWC-1.

Various types of misalignments can occur during practical honeycomb assembly processes. To compare the effects of the different misalignments, here, we studied the different assembly misalignments obtained by varying the misalignment distance between the upper and lower honeycombs, designated as “CWC-x”, where “x” indicates the misalignment distance. For example, “CWC-1/2” indicates a misalignment where one honeycomb layer is shifted by half the length of a honeycomb cell edge along the L direction. Therefore, the previously mentioned CW and CWC groups are now “CWC-0” and “CWC-1”, respectively. Different misalignment lengths affected the support length between the double-layer walls of the upper and lower honeycombs. Figure 17 shows a top view of the honeycomb structures assembled with different misalignment distances. The entire simulation group included samples ranging from fully aligned to misaligned by 1.5 times the honeycomb-cell edge length. Four sample points were collected within one assembly unit cycle to compare the performance.

#### 4.3.1. Force–Time Curves and Force–Deformation Curves after Impact

**Figure 18:**
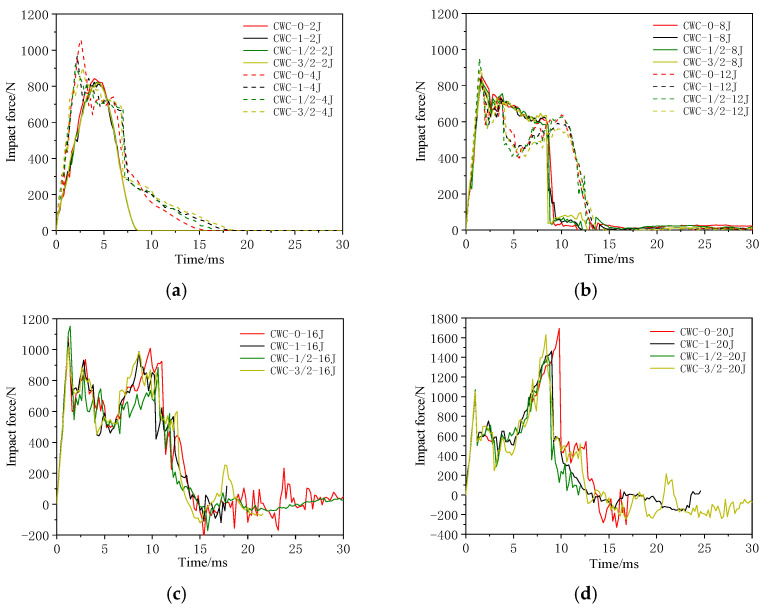
Impact force–time curves of honeycombs under different impact energies: (**a**) 2 and 4 J; (**b**) 8 and 12 J; (**c**) 16 J; (**d**) 20 J.

**Figure 19:**
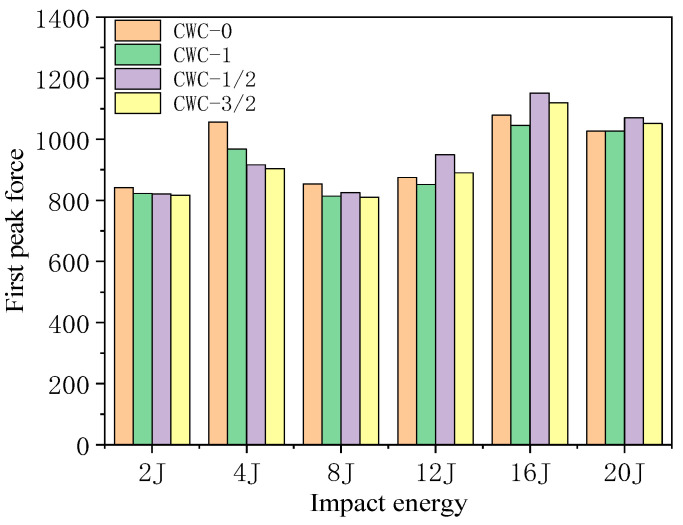
First peak of honeycomb structures under different energy levels.

Figure 18 shows the impact force–time curves for different misalignment distances. Figure 19 shows the first peak force under different impact energy levels. Finite element simulation analysis revealed that under a low-impact energy (0–8 J), the impact is not sufficient to penetrate the upper panel, and different misalignment distances slightly affect the force–deformation curve. Hence, the impact performance of the structure is not significantly affected. Combined with the peak force data of the honeycomb structures under different impact energies, shown in Table 15, the peak force of the CWC-0 honeycomb was always higher than those of the other misaligned honeycombs. Such a result implies that under low-energy impacts, the upper panel of the CWC-0 honeycomb exhibits the highest resistance. As the impactor can penetrate the upper panel, the advantage of the upper panel of the aligned honeycomb regarding impact resistance disappears. However, the first peak force curves of the four configurations nearly coincide. Subsequently, the peak force of CWC-1/2 exceeded those of the other types of honeycombs, followed by CWC-3/2, indicating that the resistance capacity of the first panel increased with misalignments of 1/2 and 3/2. However, the declining trend of the curves after reaching the peak force was steeper for these misaligned honeycombs, and the load levels after the impactor penetrated the core were CWC-1/2 < CWC-3/2. Therefore, misalignments of 1/2 and 3/2 improved the resistance capacity of the first panel but weakened the impact resistance of the core.

Before reaching the second peak force, the curve trends for all the types remained consistent; however, CWC-0 required more time to reach the second peak than the misaligned honeycombs. This implies that the impactor reaches the lower panel more quickly in misaligned honeycomb structures during the impact process. The second peak force of CWC-0 was always greater than that of the other types of honeycombs, indicating that misalignment reduces the impact resistance performance of a tandem honeycomb structure. The second peak force of CWC-1/2 was the lowest, and the impactor spent the least time in the CWC-1/2 structure, breaking through the second panel faster, indicating that CWC-1/2 exhibited the worst impact resistance. Therefore, the structural misalignment affects the resistance capacity of the second panel.

**Figure 20:**
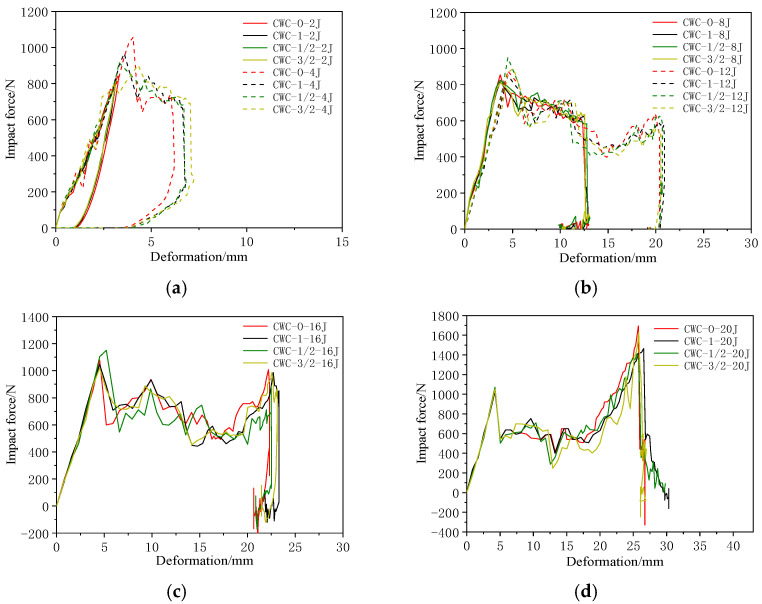
Impact force–deformation curves of honeycombs under different impact energies: (**a**) 2 and 4 J; (**b**) 8 and 12 J; (**c**) 16 J; (**d**) 20 J.

**Figure 21:**
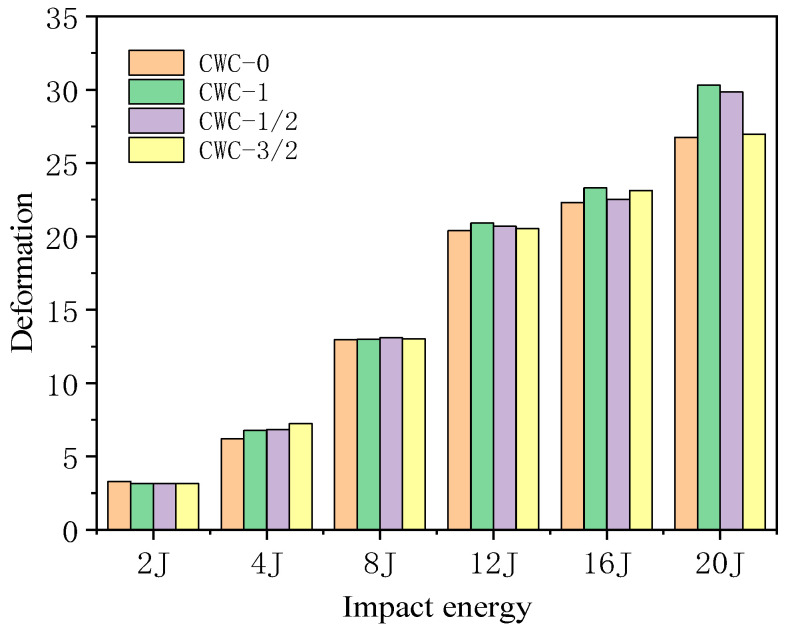
Deformation of honeycomb structures under different energy levels.

Figure 20 shows the force–deformation curves for different misalignment distances under various impact energies; the results are listed in Table 16. Figure 21 shows the deformation under different impact energy levels. The maximum impact displacement of the CWC-0 honeycomb was always less than those of the other misaligned honeycombs, indicating that the upper panel of the aligned honeycomb had a stronger resistance capacity than the misaligned honeycomb. This result is consistent with the results obtained from the force–time curves. When the impact energy was low and insufficient to penetrate the first panel, the different misalignment distances slightly affected the force–deformation curves and did not significantly affect the structural impact performance. As the impact energy increased, the CWC-1 structure required the largest displacement to dissipate the energy of the impactor, whereas the displacements required by CWC-3/2 and CWC-1/2 fell between those of these configurations.

Misalignment reduces the impact mechanical response characteristics of tandem honeycomb structures. The CWC-0 honeycomb exhibited the best impact resistance. The CWC-1 honeycomb required the largest displacement to dissipate the impactor’s energy, whereas the CWC-1/2 and CWC-3/2 honeycombs weakened the core’s impact resistance and reduced the second peak force. Thus, the misalignment distance adversely affected the structural impact of the mechanical response.

#### 4.3.2. Energy-Absorption History

**Figure 22:**
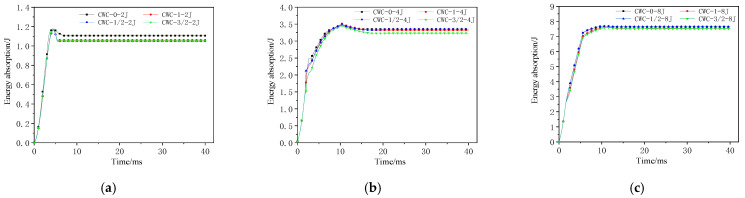
Energy-absorption–time history curves of honeycomb structures at low-energy levels: (**a**) 2 J; (**b**) 4 J; (**c**) 8 J.

**Figure 23:**
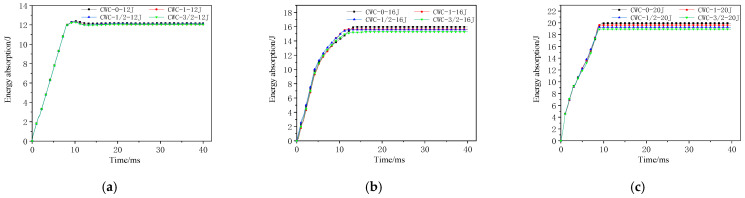
Energy-absorption–time history curves of honeycomb structures at high-energy levels: (**a**) 12 J; (**b**) 16 J; (**c**) 20 J.

**Figure 24:**
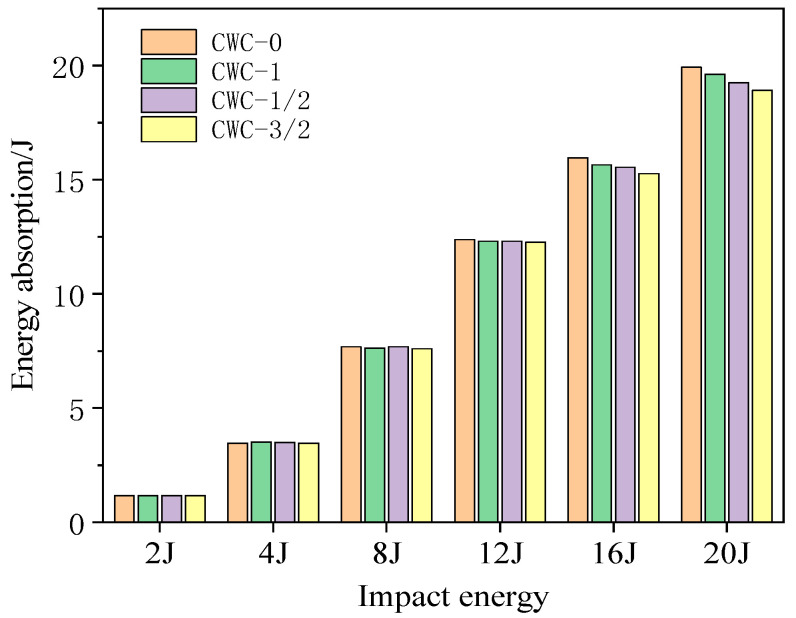
Energy absorption of honeycomb structures under different energy levels.

Figure 22 and Figure 23 show the energy-absorption curves for different misaligned honeycomb structures under impact. Figure 24 shows the energy under different impact energy levels. The misalignment distance did not significantly affect the energy-absorption rate of the honeycomb structures. The CWC-0 honeycomb always absorbed more energy than the other honeycombs, aligning with the earlier conclusion that CWC-0 has the best impact resistance. Misalignment reduces the impact mechanical response characteristics of tandem honeycomb structures. The energy-absorption curves under a high-energy impact are shown in Figure 21. At an impact energy of 12 J, the curves for all honeycomb types were consistent. As the energy increased to 16 and 20 J and the impactor contacted the lower panel, CWC-0 always absorbed the highest amount of energy and required the longest time to reach the maximum energy, indicating that CWC-0 had the highest energy-absorption level. Thus, it delayed the impactor’s penetration into the structure. Moreover, the CWC-0 honeycomb had the best impact resistance among all the types. CWC-1 followed in energy absorption, whereas CWC-3/2 absorbed less energy than CWC-1/2. Therefore, CWC-3/2 and CWC-1/2 deteriorate the energy-absorption capacity of the structure, and the misalignment distance adversely affects the mechanical response of the structure.

## 5. Conclusions

This study involved impact experiments on double-layer Nomex honeycomb sandwich structures and single-layer Nomex honeycomb sandwich structures. In addition, finite element simulations were performed for honeycomb structures with different misalignment distances. The analysis of the effects of different misalignment distances on the impact performance of honeycomb sandwich structures revealed that misalignment distances impact performance in different ways. In particular, the results provide insights into the effect of misaligned honeycomb cores on the impact mechanical responses. The replacement of metallic materials with composite panels and Nomex honeycomb cores showed good agreement with the test results. These findings can help optimize the design parameters to improve structural performance. Moreover, the quantitative metrics provided in this study, such as the peak force and displacement variations under different misalignment configurations, can serve as benchmarks for future studies considering post-impact compression performance or material fatigue properties. The main conclusions are as follows.

(1) At lower energy levels, the impact contact force exhibited a consistent linear increase. As the energy increased without reaching the penetration stress of the panel, the contact between the impactor and panel lasted longer before rebounding, resulting in a plateau phase in the stress curve. When the impact energy reached the penetration stress of the panel, a step appeared in the descending phase of the contact force, and the overall contact time was longer.

(2) Under the same energy, the double-layer honeycomb structures provided stronger resistance and less deformation, resulting in less irreversible damage to the panel and improved impact resistance, significantly outperforming the single-layer honeycomb structures. Among the double-layer honeycomb structures, the aligned honeycomb cores exhibited better impact resistance than the misaligned cores. The misaligned structures required larger impactor displacements to fully absorb the impact energy, and the impactor reached the lower panel more rapidly, indicating that the misalignment reduced the impact resistance of the tandem honeycomb structures.

(3) Regarding low-energy impacts, the CWC-0 group always exhibited a higher peak force than the other misaligned honeycomb structures. Furthermore, the CWC-0 group demonstrated a larger peak force than the other groups, approximately 3% at 2 J and 9% at 4 J, indicating its great resistance. The misalignment distance minimally affected the force–deformation curves and overall impact performance at low energies.

(4) As the energy increased, the upper panel was penetrated. The first peak force of misalignments of 1/2 and 3/2 was 5% and 4% larger than the other groups, indicating the improved resistance of the first panel. However, the impact resistance of the core was weakened, and the second peak force was reduced, thereby compromising the impact resistance of the second panel. The second peak force for CWC-0 was always higher than those for the other configurations, being 14% higher than CWC-1/2 and 2% higher than CWC-1 and CWC-3/2. The CWC-1 configuration required the largest displacement to absorb the impact energy, whereas CWC-0 required the least displacement. Those of CWC-3/2 and CWC-1/2 were in between these two configurations.

(5) The impactor reached the lower panel of the CWC-1/2 configuration in a shorter time, whereas the CWC-0 configuration required more time, delaying the impact on the lower panel. This indicates that the 1/2 misalignment results in the lowest impact resistance. Similarly, CWC-0 absorbed the highest amount of energy, followed by CWC-1, whereas CWC-3/2 and CWC-1/2 absorbed the least. Overall, the CWC-0 group demonstrated the best impact resistance across various impact energies. In contrast, misalignment reduced the impact resistance and energy-absorption capacities of the tandem honeycomb structures, thereby adversely affecting their impact mechanical responses.

This study provides practical guidance for industries requiring improved impact resistance, such as the aviation, automotive, and packaging industries. Although aligned cores in double-layer honeycomb structures exhibited superior impact resistance to misaligned cores, optimizing the misalignment distance can still provide acceptable performance levels when a complete alignment is not possible. Furthermore, at high energy levels, greater misalignment can even result in a stronger impact resistance of the upper panel. This characteristic can guide various industries in optimizing the design and assembly of honeycomb structures to enhance their mechanical performance under impact conditions, thereby improving safety, reliability, and effectiveness in specific applications.

## Figures and Tables

**Table 1:** Comparative analysis regarding impact studies on honeycomb structures.

Literature Number	Research Content and Findings	Progress/Difference
[39]	The excellent energy-absorbing properties of the double-layer honeycomb were verified by axial compression tests, and the plastic energy dissipation within the transitional deformation zone at the densification stage, as well as the influence of the strength gradient of each segment on its collapse sequence, were investigated.	The study did not evaluate the effect of the panel, which was mainly focused on the performance under axial compression, and only studied aluminum honeycomb core. In contrast, this study focused on the mechanical properties of the Nomex honeycomb complete structure under dynamic mechanical impact load.
[43]	Experimental validations of the impact response of single- and double-layer honeycomb sandwich structures with different cell sizes and core layer arrangements were performed, emphasizing the high energy-absorption capacity of double-layer structures.	References [43,45] improved and optimized the impact mechanical response of the double-layer honeycomb structure. However, these studies did not consider the effects of misaligned assemblies on the structure. Both panels and honeycomb cores were fabricated from aluminum, and the weight, designability, and other advantages of composite panels and Nomex honeycombs were not considered.
[45]	Efforts were made to reduce the maximum deflection of the front and rear panels and improve the stiffness, impact resistance and energy-absorption capacity of the structure after filling the upper and lower layers of the double-layer honeycomb sandwich structure with tubes.	
[46]	The impact response of honeycomb sandwich panels with different core types and impactor shapes was investigated. The results showed that multilayer gradient tandem honeycomb sandwich panels have the best impact-resistance performance.	In contrast to [46], this study developed finite element numerical models for the different core types.

**Table 2:** Comparison of specimen groups.

Group	Number of Core Layers	Size L × W × T/mm	Core Height/mm	Cell Side Length/mm	Side View of the Specimen
C	1	110 × 110 × 20.6	19.6	1.85	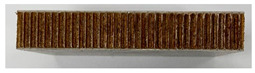
CW	2	110 × 110 × 20.6	9.6	1.85	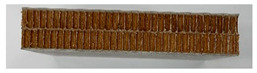
CWC	2	110 × 110 × 20.6	9.6	1.85	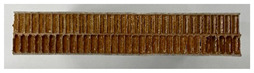

**Table 3:** Experimental parameters and results of the C group.

Number	Impact Speed m/s	Indentation Depth/mm	Diameter of Damaged Area/mm		Damage Type	Peak Force/N	
			Upper Panel	Lower Panel		First	Second
C-2 J	1.37	0.934	14.16	/	Delamination of the upper panel with minor fiber fractures	654.2	/
C-4 J	1.94	1.949	17.49	/	Extensive fiber fractures in the upper panel with visible cracking	895.4	/
C-8 J	2.74	/	27.67	/	Upper panel exhibited ±45° intersecting cracks and complete perforation	800.0	/
C-12 J	3.36	/	25.43	41.55	Upper panel was completely perforated; lower panel was also impacted	907.4	1283.7
C-16 J	3.88	/	25.36	46	Entire structure was completely perforated	1064.2	1317.2

**Table 4:** Experimental parameters and results of the CW group.

Number	Impact Speed m/s	Indentation Depth/mm	Diameter of Damaged Area/mm		Damage Type	Peak Force/N	
			Upper Panel	Lower Panel		First	Second
CW-2 J	1.37	0.367	9.73	/	Upper panel delamination	826.5	/
CW-4 J	1.94	1.701	18.73	/	Partial fiber breakage at the impact site	993.5	/
CW-8 J	2.74	2.565	27.63	/	Significant fiber breakage on the upper panel, with noticeable cracks at the impact center	853.9	/
CW-12 J	3.36	/	22.38		Complete penetration of the upper panel, with the impactor entering the core	879.1	/
CW-16 J	3.88	/	25.57	29.06	Complete penetration of the upper panel, with the lower panel showing barely visible slight bulging; impactor impacted the lower panel	1037.6	1100.7
CW-20 J	4.33	/	24.84	56.03	Complete penetration of the entire structure	945.4	1812.2

**Table 5:** Experimental parameters and results of the CWC group.

Number	Impact Speed m/s	Indentation Depth/mm	Diameter of Damaged Area/mm		Damage Type	Peak Force/N	
			Upper Panel	Lower Panel		First	Second
CWC-2 J	1.37	0.435	10.24	/	Upper panel delamination	846.6	/
CWC-4 J	1.94	1.603	17.84	/	Partial fiber breakage at the impact site	990.7	/
CWC-8 J	2.74	3.384	26.03	/	Significant fiber breakage on the upper panel, with noticeable cracks at the impact center	950.4	/
CWC-12 J	3.36	/	25.01		Complete penetration of the upper panel, with the impactor entering the core	919.2	/
CWC-16 J	3.88	/	25.06	33.08	Complete penetration of the upper panel, with the lower panel showing barely visible slight bulging; impactor impacted the lower panel	1064.3	1165.5
CWC-20 J	4.33	/	24.30	49.30	Complete penetration of the entire structure	789.5	1480.4

**Table 6:** Comparison of first peak forces under different impact energies.

Impact Energy Results	2 J	4 J	8 J	12 J	16 J	20 J
C group	654.2	895.4	800	907.4	1064.2	/
CW group	826.5	993.5	853.9	879.1	1037.6	945.4
CWC group	846.6	990.7	950.4	919.2	1064.3	789.5
Change amount of CW group	26.34%	10.96%	6.74%	−3.12%	−2.50%	/
Change amount of CWC group	29.41%	10.64%	18.80%	1.30%	0.01%	/

**Table 7:** Comparison of impact displacements under different impact energies.

Impact Energy Results	2 J	4 J	8 J	12 J	16 J	20 J
C group	3.30	6.64	18.31	23.53	32.53	/
CW group	3.10	6.10	11.71	19.31	21.66	25.36
CWC group	3.21	6.09	11.74	20.93	22.83	32.55
Change amount of CW group	−6.01%	−8.15%	−36.05%	−17.93%	−33.42%	/
Change amount of CWC group	−2.60%	−8.24%	−35.88%	−11.05%	−29.82%	/

**Table 8:** Material parameters of the honeycomb structure.

Composite Panel			Honeycomb
E_1_ = 56.1 GPa	E_2_ = 60.6 GPa	E_3_ = 10.3 GPa	E_s_ = 2643.5 MPa
G_12_ = 3460 MPa	G_23_ = 5240 MPa	G_13_ = 5240 MPa	ρ = 1143 kg/m^3^
μ_12_ = 0.04	μ_23_ = 0.26	μ_12_ = 0.26	μ = 0.3
X_T_ = 608 MPa	Y_T_ = 706 MPa	Z_T_ = 48.5 MPa	t = 0.05 mm
X_C_ = 754 MPa	Y_C_ = 706 MPa	Z_C_ = 48.5 MPa	l = 1.85 mm
S_12_ = 57.2 MPa	S_23_ = 57.2 MPa	S_13_ = 57.2 MPa	

**Table 9:** Experimental and finite element peak force data for three structures under different impact energies.

Impact Energy Results	2 J	4 J	8 J	12 J	16 J	20 J
Experiments of the C group	654.2	895.4	800	907.4	1064.2	/
FEM of the C group	646.53	949.24	869.0	984.23	1112.5	/
Error	1.17%	6.01%	8.63%	8.47%	4.54%	/
Experiments of the CW group	826.5	993.5	853.9	879.1	1037.6	945.4
FEM of the CW group	842.13	1056.65	853.992	875.238	1078.59	1026.61
Error	1.89%	6.36%	0.01%	0.44%	3.95%	8.59%
Experiments of the CWC group	846.6	990.7	950.4	919.2	1064.3	789.5
FEM of the CWC group	822.44	967.267	813.99	851.888	1044.94	1026.61
Error	2.85%	2.37%	14.35%	7.32%	1.82%	30.03%

**Table 10:** Experimental and finite element displacement data for three structures under different impact energies.

Impact Energy Results	2 J	4 J	8 J	12 J	16 J	20 J
Experiments of the C group	3.30	6.64	18.31	23.53	32.53	/
FEM of the C group	3.122	6.73	18.255	22.436	31.7636	/
Error	5.39%	1.36%	0.30%	4.65%	2.36%	/
Experiments of the CW group	3.10	6.10	11.71	19.31	21.66	25.36
FEM of the CW group	3.29	6.20	12.97	20.40	22.32	26.74
Error	6.13%	1.64%	10.76%	5.64%	3.05%	5.44%
Experiments of the CWC group	3.21	6.09	11.74	20.93	22.83	32.55
FEM of the CWC group	3.15	6.78	12.98	20.91	23.31	30.31
Error	1.87%	11.33%	10.56%	0.10%	2.10%	6.88%

**Table 11:** Damage modes of single-layer honeycombs.

Energy	SDV1		SDV2		SDV3		SDV4	
	Upper Panel	Lower Panel	Upper Panel	Lower Panel	Upper Panel	Lower Panel	Upper Panel	Lower Panel
2 J	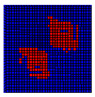		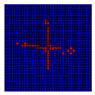		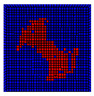		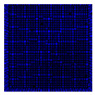	
4 J	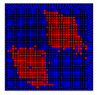		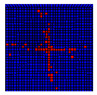		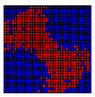		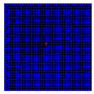	
8 J	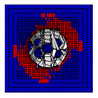		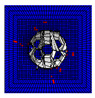		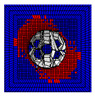		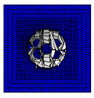	
12 J	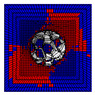	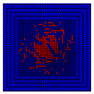	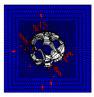	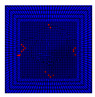	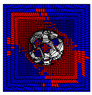	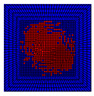	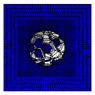	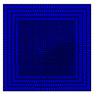
16 J	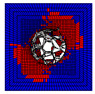	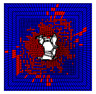	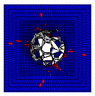	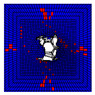	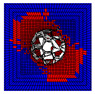	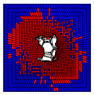	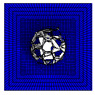	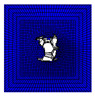

**Table 12:** Damage modes of double-layer aligned honeycombs.

Energy	SDV1		SDV2		SDV3		SDV4	
	Upper Panel	Lower Panel	Upper Panel	Lower Panel	Upper Panel	Lower Panel	Upper Panel	Lower Panel
2 J	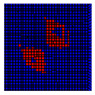		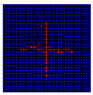		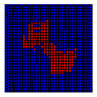		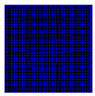	
4 J	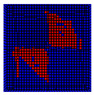		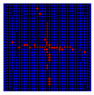		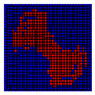		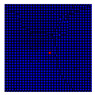	
8 J	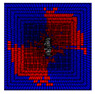		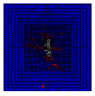		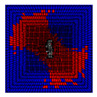		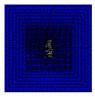	
12 J	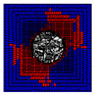		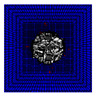		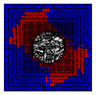		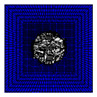	
16 J	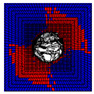	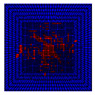	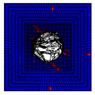	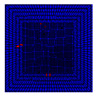	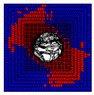	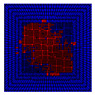	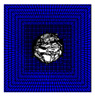	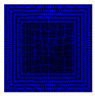
20 J	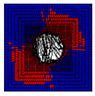	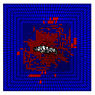	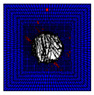	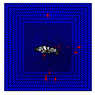	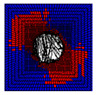	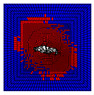	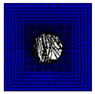	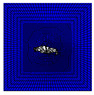

**Table 13:** Damage modes of double-layer misaligned honeycombs.

Energy	SDV1		SDV2		SDV3		SDV4	
	Upper Panel	Lower Panel	Upper Panel	Lower Panel	Upper Panel	Lower Panel	Upper Panel	Lower Panel
2 J	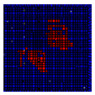		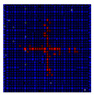		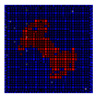		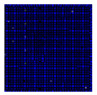	
4 J	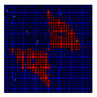		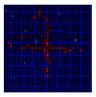		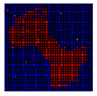		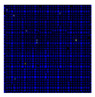	
8 J	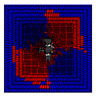		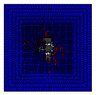		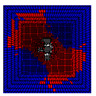		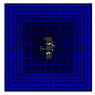	
12 J	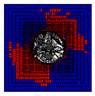		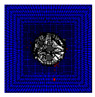		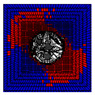		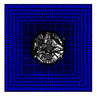	
16 J	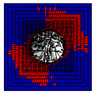	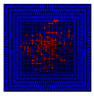	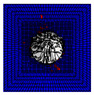	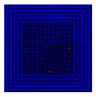	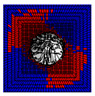	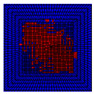	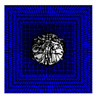	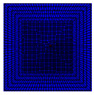
20 J	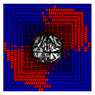	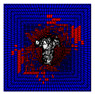	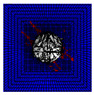	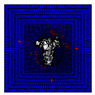	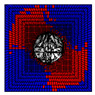	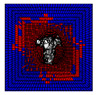	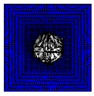	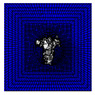

**Table 14:** Cross-sectional images of sandwich structures.

Energy	Single-Layer Honeycomb	Double-Layer Aligned Honeycomb	Double-Layer Misaligned Honeycomb
	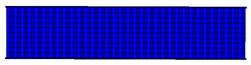	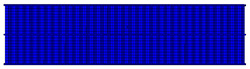	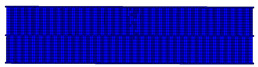
2 J	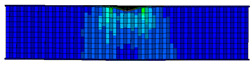	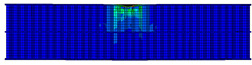	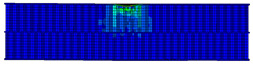
4 J	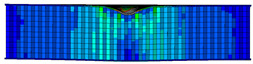	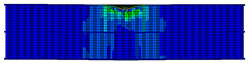	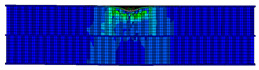
8 J	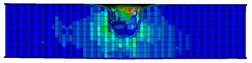	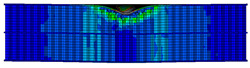	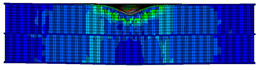
12 J	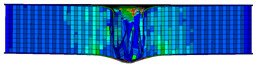	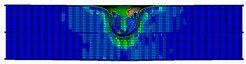	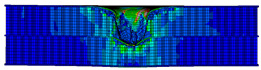
16 J	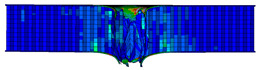	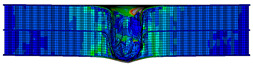	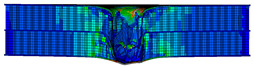
20 J		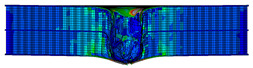	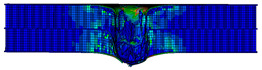

**Table 15:** Peak forces at different impact energies.

	Impact Energy	2 J	4 J	8 J	12 J	16 J	20 J
Type							
CWC-0	First	842.13	1056.65	853.99	875.24	1078.59	1026.61
CWC-1/2		821.22	916.35	824.87	949.72	1151.09	1070.91
CWC-1		822.44	967.27	813.99	851.89	1044.94	1026.61
CWC-3/2		816.35	903.21	810.19	889.72	1119.49	1051.93
CWC-0	Second	/	/	/	/	1009.20	1693.79
CWC-1/2		/	/	/	/	884.43	1409.29
CWC-1		/	/	/	/	988.20	1463.69
CWC-3/2		/	/	/	/	985.44	1630.43

**Table 16:** Displacement at different impact energies.

	Impact Energy	2 J	4 J	8 J	12 J	16 J	20 J
Type							
CWC-0		3.29	6.20	12.97	20.40	22.32	26.74
CWC-1/2		3.14	6.82	13.08	20.68	22.52	29.83
CWC-1		3.15	6.78	12.98	20.91	23.31	30.31
CWC-3/2		3.14	7.22	13.01	20.52	23.12	26.96

## Data Availability

The data presented in this study are not available on request from the corresponding author because they are part of an ongoing study.
